# Randomized Controlled Trial of Probiotic PS128 in Children with Tourette Syndrome

**DOI:** 10.3390/nu13113698

**Published:** 2021-10-21

**Authors:** Chang-Chun Wu, Lee-Chin Wong, Chia-Jui Hsu, Chianne-Wen Yang, Ying-Chieh Tsai, Feng-Shiang Cheng, Hsiao-Yun Hu, Wang-Tso Lee

**Affiliations:** 1 Department of Pediatric Neurology, National Taiwan University Children’s Hospital, Taipei 100, Taiwan; chungjane0802@gmail.com (C.-C.W.); leechinx@hotmail.com (L.-C.W.); chwenyang@gmail.com (C.-W.Y.); 2 Department of Pediatrics, Taipei City Hospital Heping Fuyou Branch, Taipei 100, Taiwan; 3 Department of Pediatrics, Cathay General Hospital, Taipei 106, Taiwan; 4 Department of Pediatrics, National Taiwan University Hospital Hsin-Chu Branch, Hsin-Chu 300, Taiwan; jry730701@gmail.com; 5 Institute of Biochemistry and Molecular Biology, National Yang Ming Chiao Tung University, Taipei 112, Taiwan; tsaiyc@ym.edu.tw; 6 Department of Education and Research, Taipei City Hospital, Taipei 106, Taiwan; A3989@tpech.gov.tw (F.-S.C.); A3547@tpech.gov.tw (H.-Y.H.); 7 Institute of Public Health, National Yang Ming Chiao Tung University, Taipei 112, Taiwan; 8 Department of Health and Welfare, University of Taipei, Taipei 100, Taiwan; 9 Graduate Institute of Brain and Mind Sciences, National Taiwan University College of Medicine, Taipei 100, Taiwan

**Keywords:** tics, probiotics PS128, Tourette syndrome, attention deficit hyperactivity disorder, children

## Abstract

Tourette syndrome results from a complex interaction between social–environmental factors, multiple genetic abnormalities, and neurotransmitter disturbances. This study is a double-blinded, randomized controlled trial using probiotics *Lactobacillus plantarum* PS128 as an intervention to examine if probiotics improve symptoms of children with Tourette syndrome. This study enrolled children aged 5 to 18 years old who fulfilled DSM-V diagnostic criteria for Tourette syndrome. Patients were assessed before initiating the trial, at one month, and at two months after randomization. The primary outcome was evaluated by Yale Global Tic Severity Scale (YGTSS), and the secondary outcome studied the possible comorbidities in these children. The results revealed no significant difference in improvement in YGTSS between the control group and the PS128 group. As for secondary endpoints, an analysis of Conners’ Continuous Performance Test (CPT) showed improvement in commission and detectability in the PS128 group. In conclusion, although probiotics may not have tic-reducing effects in children with Tourette syndrome, it may have benefits on comorbidities such as attention deficit and hyperactivity disorder (ADHD). Further studies are needed to clarify the effects of probiotics on the comorbidities of Tourette syndrome children.

## 1. Introduction

Tourette syndrome results from a complex interaction between social–environmental factors, multiple genetic abnormalities, and neurotransmitter disturbances. People with Tourette syndrome have an 85% lifetime prevalence of psychiatric comorbidities, and most comorbidities have an onset age in childhood [1]. The most common comorbid psychiatric disorders are ADHD and OCD, followed by mood disorders, anxiety disorders, and disruptive behavior disorders [1].

The proposed pathogenesis of Tourette syndrome includes disturbance in the cortico–striatal–thalamic–cortical (mesolimbic) circuit, stronger neural activity and interregional causality throughout the motor pathway [2], reduced neuroplastic changes in control systems reflected by the reduced caudate volume over time [3], hyperresponsive spike-dependent dopamine release following stimulation [4], reduction in cerebrospinal fluid of 5-hydroxyindoleacetic acid [5], and gene polymorphism [6,7]. The therapeutic effect of antidopaminergic agents on Tourette symptoms supports the pathogenesis that neurotransmitters play an important role in Tourette syndrome [8,9]. 

Probiotics are capable of altering the brain and behaviors of the host via the gut–brain axis (GBA) [10]. The gut–brain influence has been established in animal studies [11,12], and in healthy participants [13,14]. There is also much literature addressing gut–brain bidirectional communication in adult neurological diseases such as multiple sclerosis, Parkinson’s disease, and Alzheimer’s disease [15,16,17]. Studies examining the effects of probiotics and pediatric neurodevelopmental and neuropsychiatric diseases have also been done [18]. However, the contribution of gut microbiota to neurodevelopmental or neuropsychiatric diseases is complicated, and there are many other influencing factors such as genetic, epigenetic, diet, social environment, exercise, and time frame of probiotic intervention; the mechanism behind these factors is yet fully elucidated [19]. Hence, more studies are needed to explore microbiome modulation as a possible intervention for pediatric neurodevelopment and neuropsychiatric diseases.

Probiotics PS128 has been demonstrated in animal studies to be a psychobiotic strain that can modulate the levels of neurotransmitters in the brain. In animal studies, the use of live PS128 in germ-free mice increased concentrations of dopamine, serotonin, and their metabolites in the striatum [20]. Moreover, in a recent randomized controlled trial studying the effects of PS128 on children with autistic spectrum disorder (ASD) [11], the participants’ teachers observed a decrease in tic severity in those participants who had tics, which led to the generation of this study. In addition, in a previous study on children with ASD [11], there was also improvement in attention after treatment with PS128. 

Therefore, we hypothesized that PS128 may improve symptoms in children with Tourette syndrome and its comorbidities, and we conducted a double-blinded randomized-controlled trial to examine our hypothesis. 

## 2. Materials and Methods

### 2.1. Trial Design

This is a randomized clinical trial with a double-blind, placebo-controlled design to examine if probiotic PS128 may have a positive effect on Tourette syndrome children. The trial was conducted at a single tertiary care hospital in Taiwan. The Institutional Review Committee of National Taiwan University Hospital approved the study protocol. This study abides by the principles of the Declaration of Helsinki [21]. Patients or their guardians provided written informed consent. Our clinical trial was registered on ClinicalTrials.gov under the registration number NCT03259971.

### 2.2. Participants

The participants were enrolled from the Department of Pediatrics, National Taiwan University Children’s Hospital, from 1 August 2017 through to 31 January 2019. The inclusion criteria were children aged 5 to 18 years old, diagnosed with Tourette Syndrome based on the Diagnostic and Statistical Manual of Mental Disorders (5th ed.; DSM-V; American Psychiatric Association, 2013) [22]. 

The exclusion criteria included moderate to severe intellectual disability and the consumption of antibiotics and yogurt or probiotic products two weeks prior to enrollment. Participants who had moderate to severe intellectual disability based on full scale intelligence quotient scores (FSIQ < 50) [23], which was done after enrollment, were excluded from data analysis. Participants were allowed to continue their regular Tourette or ADHD medications but without alteration in medication and dosage during the trial. Participants were on their regular diet but were asked to refrain from consuming yogurt or other probiotic products during the study period. Written informed consent was obtained from all subjects and the parents or caregivers of the subjects prior to the start of the study.

### 2.3. Randomization and Blinding

**Figure 1:**
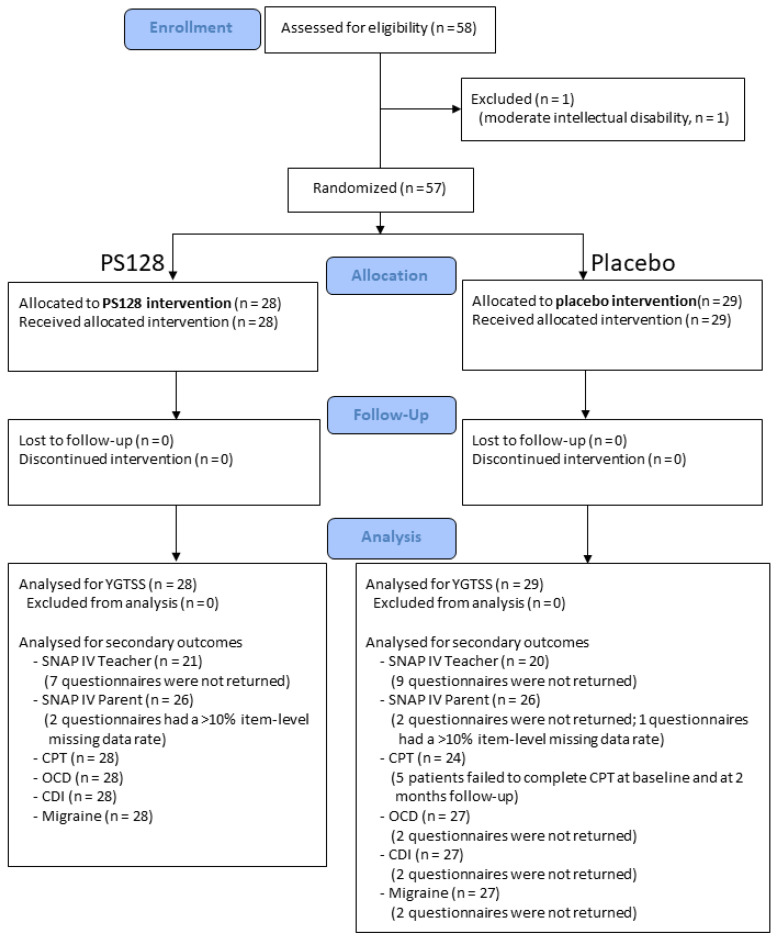
CONSORT diagram of the randomization and follow-up of the study participants.

After complying with the inclusion and exclusion criteria, the study participants were randomly assigned to 2 groups in a 1:1 ratio (Figure 1), with a block size of 4. One group received PS128, and the other received a placebo that contained microcrystalline cellulose. The PS128 and placebo capsules were identical in appearance.

Randomization was performed with a computer-generated sequence by a study coordinator who had no contact with the participants. 

The participants and their caregivers and treating physicians and the research team assessing outcomes were all masked to the group assignment. Allocation codes were disclosed only after the entire clinical trial was completed.

### 2.4. Intervention

In this clinical trial, *Lactobacillus plantarum* PS128 was used as an intervention. PS128 and placebo capsules were provided by Bened Biomedical Co., Ltd. (Taipei, Taiwan) *Lactobacillus plantarum* PS128 was isolated and deposited under DSMZ Accession No. DSM 28632 [24]. The genome architecture of PS128 has been reported previously [25]. The PS128 capsules contained 3 × 10^10^ CFU/capsule of PS128, with microcrystalline cellulose as the carrier. The placebo capsules contained only microcrystalline cellulose. All capsules were identical in taste and appearance and were stored at low temperature (4–8 °C). The capsules were given to the family on a monthly basis at each visit, and participants were asked to take one capsule 2 times a day.

### 2.5. Outcomes

Patients were randomly assigned to the placebo group or the PS128 group for 2 months. Patients were assessed before initiating the trial and at one and two months after randomization. Pediatric neurologists experienced with Tourette syndrome performed the assessment. The primary outcome, tic severity, was evaluated using the Yale Global Tic Severity Scale (YGTSS) through direct assessment by the physician. In addition, we recorded a 3–5 min video clip of the patients on each visit if the patients consented. The secondary outcome studies the possible comorbidities in these children, including attention deficit hyperactivity disorder (ADHD), obsessive-compulsive disorder (OCD), migraine, and depression. Secondary outcomes were assessed before and after the 2-month trial. 

#### 2.5.1. Tic Severity—Yale Global Tic Severity Scale (YGTSS)

YGTSS is a tool used to quantify the severity of tic symptoms in individuals aged 6–17 and is currently one of the most commonly used tools in tic assessments [26]. The YGTSS is made up of a semi-structured interview, followed by a questionnaire where individuals are asked to rate the severity of their tic symptoms (both motor and vocal) in domains such as: number, frequency, intensity, complexity, and interference [27]. The sum of the domains is termed the YGTSS total score. There is also an impairment scale, where the individual rates how the tics impact their daily life and activities [27]. Adding the impairment scale to the YGTSS total score brings about the YGTSS global score.

#### 2.5.2. ADHD—SNAP-IV Parent and Teacher Evaluation

The Swanson, Nolan, and Pelham IV Scale (SNAP-IV), a 26-item rating scale, is used to assess ADHD and oppositional defiant disorder (ODD) symptoms of children aged 6–18 years old. It consists of three subsets: attention deficit (9 items), hyperactivity disorder (18 items), and oppositional and defiance problems (8 items). The questionnaire is evaluated by parents and teachers for a more comprehensive understanding of the children’s condition. It is based on a 4-point rating scale, 0–3 points, to describe the degree to which the behavior is abnormally frequent and severe compared to same-age typically developing children. The psychometric property of the Chinese version of the SNAP-IV has been established [28,29], and it has been widely used in ADHD research in Taiwan. Participants who had more than 10% missing values on the scale were removed from further analyses [30].

#### 2.5.3. ADHD—Conners’ Continuous Performance Test II (CPT-2)

The Conners’ Continuous Performance Test II (CPT-2; Conners) is a computer-administered test designed to assess problems with attention in patients aged 6 to 18 years old [31]. The program measures participants’ reactions, and the following variables are analyzed: omissions T-score, commission T-score, hit reaction time, hit reaction time standard error, variability, detectability, response style, and perseverations. There was 1 patient who was 5 years old and not included in CPT analysis.

#### 2.5.4. OCD—Obsessive-Compulsive Inventory-Revised (OCI-R)

The Obsessive-Compulsive Inventory-Revised (OCI-R) was used to evaluate the symptoms of obsessive-compulsive disorder [32]. It is an 18-item questionnaire, with points from 0 to 4 for each item. The higher the points, the more severe the symptom is. Total scores were used for analysis. The developers of the Mandarin translation of OCI-R recommend a cutoff score of 21, meaning that those whose total score on the OCI-R is 21 or more probably have OCD [33].

#### 2.5.5. Migraine—Migraine Disability Assessment (MIDAS)

Migraine was assessed using the MIDAS (Migraine Disability Assessment) questionnaire, Taiwan version [34]. The MIDAS questionnaire is a 7-item screening tool. Participants report on the number of days they have had headaches that affected their daily life in the past 3 months. The number of days in the answers to the first 5 items are put together to measure the impact the patients’ headaches have on their lives.

#### 2.5.6. Depression—Children’s Depression Inventory, Taiwan Version (CDI-TW)

Depression was evaluated using the Children’s Depression Inventory, Taiwan version (CDI-TW). There are 27 items, and each item has three similar descriptive options reflecting the degree of the symptom, ranging from 0 to 2. According to normal population statistic results, depression disorder should be diagnosed in those with a T-score more than 65, which is equivalent to a score of 23 points in children aged 6 to 12 years old, and 25 points for adolescents aged 12 to 16 years old [35]. Total scores were used for analysis.

### 2.6. Sample Size

This study had a double-blind, randomized, parallel, placebo-controlled design. Randomization was performed upon confirmation of the inclusion and exclusion criteria.

### 2.7. Statistical Analysis

Baseline patient characteristics were compared between the PS128 group and the placebo group. Non-normal distributions were analyzed using nonparametric Wilcoxon rank-sum test statistics, and normal distributions were analyzed using two-sample *t*-tests. Fisher’s exact test was used for categorical variables. For the primary outcome, a paired *t*-test was used to analyze the YGTSS total and global score for the same subject, separated by time. The change in score and the % of change in score were analyzed. A generalized estimating equation (GEE) was also performed because the primary outcome was assessed at baseline, 1 month, and 2 months. The 95% confidence intervals (CIs) were reported to show the strength and direction of these associations. 

For secondary outcomes, a paired *t*-test was used to analyze the variables for the same subject, separated by time. Statistical significance was set at 5%, and all analyses were conducted using the SAS 9.4 statistical software package (SAS Institute, Inc., Cary, NC, USA).

## 3. Results

### 3.1. Participant Flow and Recruitment

The CONSORT diagram of the randomization and follow-up of the study participants is shown in Figure 1. We recruited 58 children who fulfilled the inclusion criteria. One patient had a moderate intellectual disability with an FSIQ score of 49 points and was excluded from data analysis due to difficulty in answering subjective questionnaires. No patients dropped out of the 2-month clinical trial.

### 3.2. Baseline Data

Patient characteristics are shown in Table 1. There were no significant differences between the two groups, except for oppositional behavior scores from the SNAP questionnaire. Regarding the prevalence of comorbidity in our study group, 22.8% had ADHD based on scores in both their SNAP-IV teacher and parent questionnaires; 17.5% had OCD based on their OCI-R scores; 7.0% had depression based on their CDI scores. There were 11 out of the 57 children who had had a headache in the past 3 months.

### 3.3. Outcomes

#### 3.3.1. YGTSS

The PS128 intervention group had a baseline YGTSS total score of 17.8 and a global score of 29.9. The placebo group had a baseline YGTSS total score of 20.6 and a global score of 33.5 (Table 1). There was no difference in the baseline severity of the two groups. Both PS128 and placebo groups showed improvement in the YGTSS total score after 2 months: −2.1 (*p*-value = 0.046) and −3.2 (*p*-value = 0.005), respectively, in terms of score (Table 2), and −15.0% (*p*-value = 0.043) and −13.7% (*p*-value = 0.015), respectively, in terms of percentage of change (Table 3). Both groups showed improvement after the trial, but PS128 was not superior to placebo. As for the YGTSS global score, both groups showed improvement, which was −4.6 (*p*-value = 0.156) in the PS128 group and −7.6 (*p*-value = 0.019) in the placebo group. There was a 14.2% (*p*-value = 0.215) reduction in the PS128 group, and a 20.6% (*p*-value = 0.004) reduction in the placebo group. A generalized estimating equation (GEE) was also done to account for correlations between binary outcomes across time within the same individual (Table S1). It showed no superior effects of PS128 over placebo over time.

#### 3.3.2. SNAP-IV

SNAP-IV scores were analyzed by the difference in scores over 2 months by paired *t*-test (Table 4). SNAP-IV parent and teacher questionnaires were analyzed separately. There were significant reductions in SNAP-IV scores from parents’ evaluation in the intervention group, including total score (−3.9; *p*-value = 0.021), inattention score (−2.0; *p*-value = 0.005), and hyperactivity/impulsivity (−1.8; *p*-value = 0.027) in the PS128 group. This improvement was not seen in the control group. However, both groups did not show significant improvements from teachers’ evaluations.

#### 3.3.3. CPT

The change of the CPT parameters after treatment in each group are shown in Table 5. One 5-year-old patient who belonged to the placebo group was excluded from CPT analysis because the software was designed and validated for those above 6 years old. Five patients’ CPT data from the placebo group could not be analyzed due to invalid scores either at baseline (*n* = 3) or at 2 months (*n* = 1) or both (*n* = 1). As shown in Table 5, there was a significant improvement in the commission *t* score and detectability of the PS128 group but not in the placebo group.

#### 3.3.4. CDI

A paired *t*-test was performed for CDI and showed reduced scores (−3.78 +/− 4.91, *p*-value = 0.001) in the placebo group (Table S2).

#### 3.3.5. OCI-R

A paired *t*-test was performed for OCI-R, and neither group showed significant improvement (Table S2).

#### 3.3.6. MIDAS

A paired *t*-test was performed for MIDAS, and neither group showed significant improvement (Table S2).

## 4. Discussion

The present study reflected that intervention with PS128 did not have a superior response in tic severity improvement compared to the control group. However, there was a significant improvement in some parameters of the most common comorbidity of Tourette syndrome patients, ADHD. This might suggest a possible beneficial effect of PS128 in children with Tourette syndrome.

The primary outcome, YGTSS, improved in both groups, and PS128 did not demonstrate a superior response. There are a few possible explanations. Firstly, PS128 has been demonstrated in germ-free mice to increase concentrations of dopamine, serotonin, and their metabolites in the striatum [20]. However, it is known that patients with Tourette syndrome may have dopamine hypersensitivity and, therefore, respond to dopamine blocking agents such as risperidone, pimozide, and haloperidol [36]. This correlation has also been supported by functional imaging and postmortem studies [4,37]. Therefore, it may be explainable that the use of PS128 did not show a superior response in improving tic severity when compared to the control group. Secondly, the improvement seen in both groups may reflect the wax and wane characteristics in Tourette syndrome. Thirdly, it is possible to attribute the improvement to bias by physicians who did the assessment. 

In our study, we assessed the comorbidity of ADHD in children using the SNAP-IV questionnaire from both teachers and parents. The SNAP-IV questionnaire showed reduced scores in the intervention group from the parents’ perspective but no improvement from the teachers’ point of view. This may be partially due to a change in the teacher answering the assessment if the participants had gone on to another school year; alternatively, some teachers may not have paid attention to the change in the students. In our study, the CPT test was also performed to evaluate the change in the parameters in ADHD. We found that the intervention group showed improvement in commission *t* score and detectability parameters (Table 5). However, the control group did not show any improvement. The pathophysiology behind selective attention involves the cholinergic and dopaminergic systems [38]. Current evidence also suggests that ADHD symptoms may be caused by the reduction of two other neurotransmitters: norepinephrine and serotonin [39]. There are known benefits of noradrenergic drugs in ADHD [40]. As PS128 increases dopamine and serotonin levels in germ-free mice, an increase in brain dopamine might explain our findings in improved ADHD symptoms. Whether PS128 also alters noradrenergic levels may need further study to clarify. ADHD is as troubling, if not more, as tics in school learning. Although most children with Tourette syndrome do not need treatment due to mild symptoms, the severity of ADHD in these children may need medications to improve attention. Our findings support that PS128 may be beneficial for improving the ADHD-related symptoms assessed by the SNAP-IV parent form and CPT for children with Tourette syndrome (Table 3 and Table 4) and may avoid the known side effects from taking ADHD medications.

If serotonin is partially the cause of improvement in ADHD symptoms, there should be an improvement in OCD symptoms as OCD is related to serotonin [41]. This was not reflected in our study. In our study, there were few patients with OCD, most likely due to the age group of our participants. It is consistent with the epidemiology of OCD in childhood, with the onset of their OCD symptoms in late childhood or early adolescence [42]. 

Neither did PS128 cause reduced scores in CDI. This is most likely due to the low CDI scores in our participants. Most of our participants also did not have migraines at all. The mean MIDAS score was 0.37 and 0.59 in either group. One participant in the intervention group had a MIDAS score of 120, and this decreased to 0 after being prescribed with flunarizine; this participant was subsequently left out of the MIDAS analysis. 

To our knowledge, this study is the first clinical trial that has used probiotics in randomized, double-blind, and placebo-controlled settings to assess its effect on Tourette syndrome severity and its comorbidities. There were no adverse effects reported. 

There were some limitations to the present study. The evaluations were assessed by three physicians. Although there is no discrepancy between the ratings performed, a consensus was achieved through the principal investigator, and video recordings were collected and reviewed when in uncertainty. This is the first study to investigate the effects of PS128 on children with Tourette syndrome, so the sample size was not calculated based on the improvement of outcome measured, effect size, or errors for type I and type II. Another limitation of this study is that Tourette severity often waxes and wanes. Hence, a longer study duration would provide us with a better understanding of the effects of probiotics. A weakness of our study is that we did not use biomarkers such as dopamine and serotonin levels to do correlations. We considered blood drawing too invasive in the pediatric population and that this may affect trial recruitment and retention. Another is that knowing that physical activity may also contribute to the interaction of microbiome and neurotransmitter [14], we did not include the participants’ level of physical activity in the study design. Noteworthy, there is a positive skew in our age group. Therefore, our results may be generalized to other Tourette children, particularly those yet in their teens. 

## 5. Conclusions

In summary, this study has demonstrated that probiotics PS128 does not affect children’s tic severity but improves ADHD symptoms. Our study demonstrates that two months of use of probiotics PS128 treatment improved Tourette children’s SNAP-IV (parent) and CPT scores. PS128 may be an appealing adjunctive therapy for Tourette children who have ADHD without adverse effects. Larger and longer trials should be done to fully understand the effect of probiotics on Tourette children’s behavior. 

## 6. Patents

A patent is under application.

## Figures and Tables

**Table 1:** Patient characteristics, baseline YGTSS, and comorbidity assessments.

Variable	PS128 (N = 28)		Placebo (N = 29)		
	N	Median (Q1, Q3) or n (%)	N	Median (Q1, Q3) or n (%)	*p*-Value
**Age**	28	9.3 (8.5, 10.3)	29	10.4 (8.7, 12.2)	0.139
**Gender**					
Male	28	24 (85.71)	29	24 (82.76)	0.999 ^a^
Female		4 (14.29)		5 (17.24)	
**IQ**	28	102.0 (95.0, 113.0)	29	103.0 (96.0, 108.0)	0.546
**Medication use**					0.346 ^a^
No	28	11 (39.30)	29	15 (51.70)	
Yes		17 (60.70)		14 (48.30)	
**Medication type**					
Aripiprazole	28	16 (57.14)	29	12 (41.38)	0.234 ^a^
Biperiden	28	1 (3.57)	29	0 (0)	0.491 ^a^
Clonazepam	28	4 (14.29)	29	1 (3.45)	0.194 ^a^
Clonidine	28	2 (7.14)	29	2 (6.90)	0.999 ^a^
Methylphenidate	28	1 (3.57)	29	0 (0)	0.491 ^a^
Risperidone	28	1 (3.57)	29	0 (0)	0.491 ^a^
Sulpride	28	0 (0)	29	1 (3.45)	0.999 ^a^
Other	28	1 (3.57)	29	1 (3.45)	0.999 ^a^
**YGTSS**					
YGTSS Total	28	18.0 (13.0, 22.0)	29	19.0 (13.0, 26.0)	0.460
YGTSS Global	28	27.0 (18.5, 39.5)	29	30.0 (19.0, 46.0)	0.634
**SNAPIV**					
Teacher					
Total	23	11.0 (6.0, 20.0)	28	14.5 (7.7, 26.5)	0.281
Inattention	23	8.0 (2.0, 12.0)	28	7.0 (5.0, 13.5)	0.540
Hyperactivity/Impulsivity	23	3.0 (1.0, 5.0)	28	3.0 (1.5, 7.0)	0.299
Oppositional behavior	23	1.0 (0.0, 3.0)	28	3.0 (0.5, 4.5)	0.160
Parent					
Total	25	24.0 (22.0, 32.0)	28	26.5 (18.0, 38.5)	0.187 ^b^
Inattention	25	10.0 (7.0, 14.0)	28	10.0 (6.0, 15.5)	0.845
Hyperactivity/Impulsivity	25	6.0 (4.5, 11.0)	28	9.0 (4.5, 11.5)	0.333 ^b^
Oppositional behavior	25	6.0 (4.0, 8.0)	28	9.5 (5.0, 12.0)	0.039 *
**CPT**					
Omissions T-score	28	45.8 (43.3, 51.1)	24	45.5 (43.6, 50.9)	0.862
Commissions	28	48.4 (36.8, 53.3)	24	49.3 (41.3, 59.0)	0.326
Hit RT	28	49.4 (41.5, 60.7)	24	48.5 (38.6, 53.3)	0.159 ^b^
Hit RT Std. Error	28	49.1 (42.6, 54.5)	24	45.7 (37.2, 55.5)	0.344
Variability	28	48.0 (41.7, 53.7)	24	45.5 (39.2, 54.7)	0.611 ^b^
Detectability (d’)	28	49.9 (43.8, 55.4)	24	49.4 (44.2, 58.4)	0.452 ^b^
Response Style (B)	28	50.5 (45.2, 55.8)	24	46.4 (45.4, 52.5)	0.235
Perseverations	28	46.3 (43.8, 51.4)	24	48.0 (45.6, 55.7)	0.198
**OCI-R**					
score	28	9.0 (5.5, 12.0)	29	14.0 (5.0, 19.0)	0.307
≥21 patient number	28	4 (14.29)	29	6 (20.69)	0.730 ^a^
**CDI**	28	7.5 (4.5, 13.5)	29	12.0 (5.0, 17.0)	0.179
**MIDAS**	28	0.0 (0.0, 0.0)	29	0.0 (0.0, 0.0)	0.813

^a^ Fisher’s exact test. ^b^ Two-sample *t*-test. * *p* < 0.05. CDI: Children’s Depression Inventory; CPT: Conners’ Continuous Performance Test II; FSIQ: Full Scale Intelligence Quotient; MIDAS: Migraine Disability Assessment; OCI-R: Obsessive-Compulsive Inventory-Revised; SNAP-IV: Swanson, Nolan, and Pelham (SNAP)-IV, Taiwan version; YGTSS: Yale Global Tic Severity Scale.

**Table 2:** Paired *t* analysis of change in YGTSS total and global scores between 2 months, 1 month, and baseline.

	N	PS128 (N = 28)	*p*-Value	N	Placebo (N = 29)	*p*-Value
**1 month and baseline**						
YGTSS Total	28	−2.21 ± 4.71	0.019 *	29	−3.10 ± 7.12	0.026 *
YGTSS Global	28	−7.43 ± 16.77	0.027 *	29	−5.17 ± 13.91	0.055
**2 months and 1 month**						
YGTSS Total	27	0.11 ± 5.25	0.915	27	−0.14 ± 6.06	0.903
YGTSS Global	26	2.82 ± 12.24	0.233	27	−2.38 ± 13.09	0.336
**2 months and baseline**						
YGTSS Total	28	−2.11 ± 5.32	0.046 *	29	−3.24 ± 5.79	0.005 *
YGTSS Global	28	−4.61 ± 16.68	0.156	29	−7.55 ± 16.37	0.019 *

The results are expressed as means ± SD. * *p* < 0.05. YGTSS: Yale Global Tic Severity Scale.

**Table 3:** Paired *t* analysis of % of change in YGTSS total and global scores between 2 months, 1 month, and baseline.

	N	PS128 (N = 28)	*p*-Value	N	Placebo (N = 29)	*p*-Value
**1 month and baseline**						
%change of YGTSS Total	28	−16.31 ± 29.36	0.007 *	29	−14.93 ± 39.67	0.052 *
%change of YGTSS Global	28	−19.43 ± 46.74	0.037 *	29	−14.02 ± 41.40	0.079
**2 months and 1 month**						
%change of YGTSS Total	27	7.97 ± 59.65	0.494	27	7.79 ± 69.69	0.566
%change of YGTSS Global	26	22.58 ± 100.50	0.263	27	−13.47 ± 33.26	0.045 *
**2 months and baseline**						
%change of YGTSS Total	28	−15.02 ± 37.49	0.043 *	29	−13.66 ± 28.52	0.015 *
%change of YGTSS Global	28	−14.20 ± 59.21	0.215	29	−20.57 ± 35.78	0.004 *

The results are expressed as means ± SD. * *p* < 0.05. YGTSS: Yale Global Tic Severity Scale.

**Table 4:** Analysis of SNAP-IV teacher and parent questionnaires between 2 months and baseline.

Variable	PS128 (N = 21)	*p*-Value	Placebo (N = 20)	*p*-Value
**Teacher**				
Total	1.00 ± 7.62	0.554	−2.75 ± 9.79	0.224
Inattention	0.86 ± 4.05	0.344	−1.45 ± 4.49	0.165
Hyperactivity/Impulsivity	0.48 ± 2.91	0.462	−0.20 ± 3.86	0.819
Oppositional behavior	0.33 ± 2.18	0.491	−1.10 ± 3.28	0.150
	PS128 (N = 25)	*p*-Value	Placebo (N = 26)	*p*-Value
**Parent**				
Total	−3.88 ± 7.88	0.021 *	−3.69 ± 10.12	0.075
Inattention	−2.04 ± 3.27	0.005 *	−1.27 ± 3.21	0.054
Hyperactivity/Impulsivity	−1.76 ± 3.74	0.027 *	−1.12 ± 4.09	0.177
Oppositional behavior	−0.08 ± 3.30	0.905	−1.31 ± 4.90	0.186

* *p* < 0.05. SNAP-IV: Swanson, Nolan, and Pelham (SNAP)-IV, Taiwan version.

**Table 5:** The change of the parameters of CPT after treatment in the PS128 group and the placebo group.

Variable	PS128 (N = 28)	*p*-Value	Placebo (N = 24)	*p*-Value
Omissions T-score	0.91 ± 11.05	0.667	0.91 ± 10.69	0.681
Commissions	−4.25 ± 9.22	0.022 *	−3.02 ± 8.12	0.082
Hit RT	2.09 ± 6.99	0.126	2.61 ± 11.62	0.282
Hit RT Std. Error	2.10 ± 6.56	0.103	1.28 ± 8.50	0.468
Variability	1.84 ± 8.39	0.257	1.73 ± 9.62	0.389
Detectability (d’)	−4.71 ± 10.02	0.019 *	−4.73 ± 13.29	0.094
Response Style (B)	1.60 ± 13.93	0.548	2.75 ± 13.50	0.329
Perseverations	0.56 ± 19.10	0.879	−1.38 ± 24.00	0.781

* *p* < 0.05. CPT: Conners’ Continuous Performance Test II; RT: reaction time.

## Data Availability

The data presented in this study are available on request from the corresponding author.

## Supplementary Materials

The following are available online at https://www.mdpi.com/article/10.3390/nu13113698/s1, Table S1: YGTSS total and global score GEE analysis between 2 months, 1 month, and baseline, Table S2: Paired *t* analysis of CDI, OCI-R, and MIDAS between 2 months and baseline.
