# Supplementary material for: Randomized Controlled Trial of Probiotic PS128 in Children with Tourette Syndrome
Source: Nutrients. 2021 Oct 21;13(11):3698. doi: 10.3390/nu13113698 (PMC8619307; doi:10.3390/nu13113698)
Supplement: Supplementary file 1 [file nutrients-13-03698-s001.zip › nutrients-1415712-supplementary.pdf]

Table Supplementary S1. YGTSS Total and Global score GEE analysis between 2 months, 1month, and baseline.

Outcomes/Effect	Coefficients	95% confidence interval	*P*-value
**YGTSS Total**			
PS-128 (vs. Placebo)	-2.77	(-7.37, 1.83)	0.233
Time	-1.34	(-2.36, -0.31)	0.011*
PS-128*Time	0.57	(-1.49, 2.62)	0.586
**YGTSS Global**			
PS-128 (vs. Placebo)	-4.04	(-13.54, 5.47)	0.399
Time	-3.04	(-5.49, -0.59)	0.032*
PS-128*Time	1.47	(-3.43, 6.38)	0.553

* $P < 0.05$. YGTSS: Yale Global Tic Severity Scale.

Table Supplementary S2. Paired-t analysis of CDI, OCI-R, MIDAS between 2 months and baseline.

Variable	PS128				Placebo			
	N	mean	SD	*P*- Value	N	mean	SD	*P*- Value
CDI	28	-0.82	4.42	0.334	27	-3.78	4.91	0.001*
OCI-R	28	-1.61	5.75	0.151	27	-2.89	8.41	0.086
MIDAS	28	-0.22	1.15	0.327	27	-0.48	1.65	0.141

* $P < 0.05$. CDI: Children's Depression Inventory; MIDAS: Migraine Disability Assessment; OCI-R: Obsessive Compulsive Inventory – Revised.
